# In Vitro Trueness and Precision of Intraoral Scanners in a Four-Implant Complete-Arch Model

**DOI:** 10.3390/dj11010027

**Published:** 2023-01-12

**Authors:** Dimitrios Spagopoulos, George Kaisarlis, Foteini Spagopoulou, Demetrios J. Halazonetis, Jan-Frederik Güth, Efstratios Papazoglou

**Affiliations:** 1 Department of Operative Dentistry, National and Kapodistrian University, 11527 Athens, Greece; 2 School of Mechanical Engineering, National Technical University of Athens, 15773 Athens, Greece; 3 Independent Researcher, 10829 Berlin, Germany; 4 Department of Orthodontics, School of Dentistry, National and Kapodistrian University, 11527 Athens, Greece; 5 Poliklinik für Zahnärztliche Prothetik, Center for Dentistry and Oral Medicine, Goethe-University, 60596 Frankfurt, Germany; 6 Graduate Program of Restorative Dentistry, School of Dentistry, National and Kapodistrian University, 11527 Athens, Greece

**Keywords:** intraoral scanner, IOS, laboratory scanner, trueness, precision, accuracy

## Abstract

(1) Background: New intraoral (IOS) and laboratory scanners appear in the market and their trueness and precision have not been compared. (2) Methods: Seven IOS and two laboratory scanners were used to scan a mandibular edentulous model with four parallel internal hexagon implant analogues and PEEK scan bodies. Digital models in Standard Tessellation Language (STL) were created. The master model with the scan bodies was scanned (×10) with a computerized numerical control 3D Coordinate Measuring Machine (CMM). The short (distances of adjacent scan posts) and long distances (distances of the scan posts with non-adjacent sites in the arch) among the centroids of the four analogues were calculated using CMM special software. Trueness (comparisons with the master model) and precision (intragroup comparisons) were statistically compared with ANOVA, chi-square and Tukey tests. (3) Results: Laboratory scanners had the best trueness and precision compared to all IOSs for long distances. Only iTero (Align Technologies Inc., Milpitas, CA, USA) had comparable trueness with one laboratory scanner in short and long distances. For short distances, CS3600 (Carestream Health, Inc., Rochester, NY, USA), Omnicam, Primescan (Sirona Dental Sys-tems GmbH, Bens-heim, Germany) and TRIOS 4 (3Shape A/S, Copen-hagen, Denmark) had similar trueness to one laboratory scanner. From those, only Omnicam and Primescan had similar precision as the same laboratory scanner. Most IOSs seem to work better for smaller distances and are less precise in cross-arch distances. (4) Conclusions: The laboratory scanners showed significantly higher trueness and precision than all IOSs tested for the long-distance group; for the short distance, some IOSs were not different in trueness and precision than the laboratory scanners.

## 1. Introduction

It has been suggested that Intraoral Scanners (IOSs) can provide both the patient and the clinician with pleasant experiences with short appointments [1,2]. The use of IOSs changes the workflow for the development of dental-restoration procedures by aborting the analog impression of the conventional technique, while minimizing patient discomfort [3,4]. Regardless of the selected technique, the impression must be accurate enough to allow a well-fitting prosthesis to be made [5,6].

According to ISO specifications [7,8], accuracy is the combination of trueness and precision. Trueness describes the measurement of value of an object in comparison to the pragmatic value of that object. To determine the accuracy of an IOS, it is necessary to compare its measurements to those of a reference measurement machine that is known to be highly accurate due to its low uncertainty. For this purpose, coordinate measuring machines with certified accuracy close to 3.5 μm can provide a reference close to reality [4,9]. Precision describes the ability to achieve repeatable measurements. A reference is not needed to determine the precision of IOSs; comparing repeated measurements made with the same IOS and analyzing the differences between them is enough [4,9].

The literature supports the use of IOSs for creating short-span restorations, such as single crowns and short-fixed partial dentures [4,9], but there are very few in vitro studies supporting their use in complete-arch prostheses over analog impressions, where the cross-arch accuracy is important. Papaspyridakos et al., in a five-implant mandibular model and the superimposition method for measurement, found that open-tray impression with splinted implants had statistically lower 3D deviations than a digital impression with TRIOS 3 (3shape A/S, Copenhagen, Denmark) [10]. Adversely, in a follow-up paper from the same group, Amin, et al. suggested that two IOSs [True Definition scanner 4.1., 3M Company, St. Paul, MN, USA and CEREC Omnicam 4.4.1 (Sirona Dental Systems GmbH, Bensheim, Germany)] were more accurate to conventional complete-arch implant impressions [11]. Alikhasi et al. found that digital impressions demonstrated a superior outcome in comparison with analog impressions of non-splinted implants in an edentulous mandibular model with four implants placed between the mandible foramina [12]. Ribeiro et al. showed that digital impression (TRIOS 3, 3Shape A/S, Copenhagen, Denmark) was more accurate for parallel implants but less accurate for tilted implants [13].

Kim et al. measured trueness and precision of implant centroids of full-arch implant impressions using IOSs (TRIOS 3, 3Shape A/S, Copenhagen, Denmark) and showed that conventional implant-level impressions with open-tray and splinted implants were more accurate than digital impressions [5]. Tan et al. found that polyether consistently exhibited the best accuracy in comparison to the IOSs TRIOS 3 and True Definition at all implant locations, while True Definition exhibited the poorest accuracy for all linear distortions. Additionally, they found that reducing inter-implant distance may decrease global linear distortions for intraoral scanner systems but had no effect on polyether and the dental laboratory scanner systems [14].

It seems that different brands of IOSs can differ significantly in terms of accuracy [4,9,15] and that precision is decreased significantly with increasing distances between implants and, therefore, scan bodies [15,16]. On the other hand, laboratory scanners are quite accurate and have been used to create reference models to compare IOSs in different in vitro studies [6]. There are many factors affecting the accuracy of digital impressions: IOS hardware, software, experience of the operator, characteristics of the scan bodies and clinical factors [6]. From the factors mentioned, IOS hardware and software have changed dramatically over the last few years and it is important to update our knowledge in terms of accuracy with the newest software and hardware that exist.

Mangano et al. measured the trueness and precision for four IOSs and found values from 60.6 to 112.4 μm [CS3500 (Carestream Health, Inc., Rochester, NY, USA), TRIOS (3Shape A/S, Copenhagen, Denmark), Zfx intrascan Zfx GmbH, Tübingen, Germany)] and 204.2 to 253.4 μm for Planscan [17]. Imbrugia et al. also found values for IOSs [CS3600 (Carestream Health, Inc., Rochester, NY, USA), Omnicam (Sirona Dental Systems GmbH, Bensheim, Germany), TRIOS 3 (3Shape A/S, Copenhagen, Denmark), True Definition scanner (3M Company, St. Paul, MN, USA)] that ranged from 60.6 to 106.4 μm [18]. Mangano et al. reported values of trueness for 12 different intraoral scanners that ranged from 30.4 to 98.4 μm and reported statistically significant differences among these IOSs [4]. All researchers commented that further studies are needed to confirm these results. Sami et al. evaluated the trueness and precision of four IOSs on a six-implant model. The IOSs used [True definition (3M Company, St. Paul, MN, USA), TRIOS (3Shape A/S, Copenhagen, Denmark), CEREC Omnicam (Sirona Dental Systems GmbH, Bensheim, Germany), Emerald (Planmeca Oy, Vantaa, Finland)] were not true even 10% of the time at ±0.01 mm tolerance, although when tolerance changed to ±0.05-mm, the trueness increased dramatically and they found no difference in accuracy among the four IOSs [19].

Since new IOSs come on the market and software evolves, there is a continued need for evaluation of newer IOS models for their trueness and precision in comparison to laboratory scanners. The most demanding experimental model for comparing them is the complete-arch implant model, which is better than the complete-arch dentate model since it contains shapes of regular geometry rendering measurements easier to perform. The null hypotheses of this investigation were that there is no significant difference in the trueness and precision among several intraoral and laboratory scanners in an in vitro setting of a four-implant complete-arch model. Additional null hypotheses were that there is no difference in the trueness and precision between short and long implant distances among implants in complete-arch implant impressions.

## 2. Materials and Methods

Seven intraoral scanners (CS 3600, Carestream Dental; Emerald, Planmeca; i500, Medit; iTero Element 5D, Align Technologies; Omnicam and Primescan, Dentsply Sirona; TRIOS 4, 3Shape) and two laboratory scanners (Aadva Lab Scan, GC; Ceramill Map 600, Amann Girrbach AG) were selected to compare their trueness and precision in an in vitro setup (Table 1).

### 2.1. Study Design

A mandibular arch edentulous model made out of type III dental stone was used to drill 4 close-to-parallel sites for implant placement in the positions of right and left canines and molars [20]. Cyanoacrylate adhesive was used in the prepared sites to stabilize 4 implant analogues (internal hexagon 3.8 mm Xive, Friadent/Dentsply, Mannheim, Germany). A closed-tray impression was taken from this initial model according to the steps described below and a master cast was fabricated out of type IV stone (Fujirock EP, GC Corporation, Tokyo, Japan). Milling stainless-steel implant analogs were used for this master model (Product Number 452641, Friadent Milling Implant, Dentsply Sirona, York, PA, USA). PEEK scan bodies (Elos accurate Scan body, Elos Medtech, Kungsbacka, Sweden) with titanium hexagonal connection were used to make digital impressions and create digital models.

The master model with the scan bodies was scanned (×10) with a computerized numerical control 3D Coordinate Measuring Machine (CMM) (Mistral 070705, DEA, Brown & Sharpe, HEXAGON MI, North Kingstown, WA, USA) at the Mechanical Design and Control Systems Division, School of Mechanical Engineering, National Technical University of Athens, 3 weeks after fabrication and storage in controlled environmental conditions (20 °C, 50% humidity). Dimensional changes were observed in the first two weeks after pouring, but dimensions remained stable later [21].

The CMM has a verified performance in accordance with industry-standard verification tests as per ISO 10360-2:2009 [22] and ISO 10360-5:2010 [23]. Therefore, the maximum permissible length error (E0, MPE) of this CMM is (3.5(μm) + L(mm)/250), where L is the measured length (in mm), and the single-stylus form error (PFTU) is 3.5 μm [22,23]. The probing system of the CMM is the Renishaw PH10M motorized head with TP200 touch-trigger probe (both of Renishaw plc., Wotton-under-Edge, UK). In order to collect the required set of contact points, a 20 mm-length ruby-ball tip with diameter of 2 mm was used. The CMM-obtained point coordinates were then numerically processed by PC-DMIS CAD++ v.2020 R2, the widely used measurement software of HEXAGON MI that is certified as per ISO 10360-6 by PTB [24].

**Figure 1:**
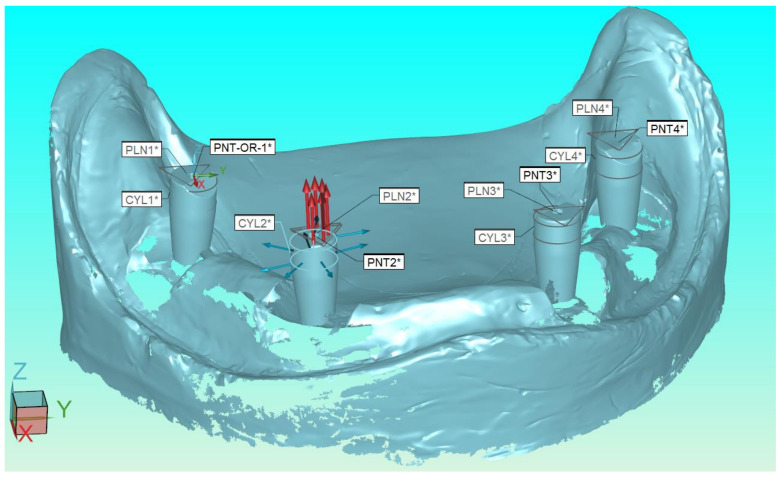
Contact points collected on the outer cylindrical surface (8 points marked in blue) and on top planar surface of the cylindrical element (6 points marked in red) for each implant in PC-DMIS measurement software.

In each implant scan body, 8 contact points were collected on its cylindrical outer surface and 6 contact points on its planar surface at the top of the cylindrical element (Figure 1). The number and distribution of all captured contact points followed the recommendations of the BS7172 standard [25].

Using standardized mathematical algorithms, the CMM special software performed the numerical fitting of cylindrical features to the sets of the 8 contact points and of planar features to the sets of the 6 contact points. The numerical intersection of the planar feature and the axis derived by the cylindrical feature in each scan body produced a “pierce point”, hereafter referred to as scan-body centroid. A number was assigned to each implant scan body (Figure 1). All scan-body centroids were located using special software and were assigned ***x***, ***y***, ***z***, coordinates and the distances between them were calculated using the formula:

$$distance_{1-2}={}^{}\sqrt[{}]{{\left(x_{1}-x_{2}\right)}^{2}+{\left(y_{1}-y_{2}\right)}^{2}+{\left(z_{1}-z_{2}\right)}^{2}}$$

**Figure 2:**
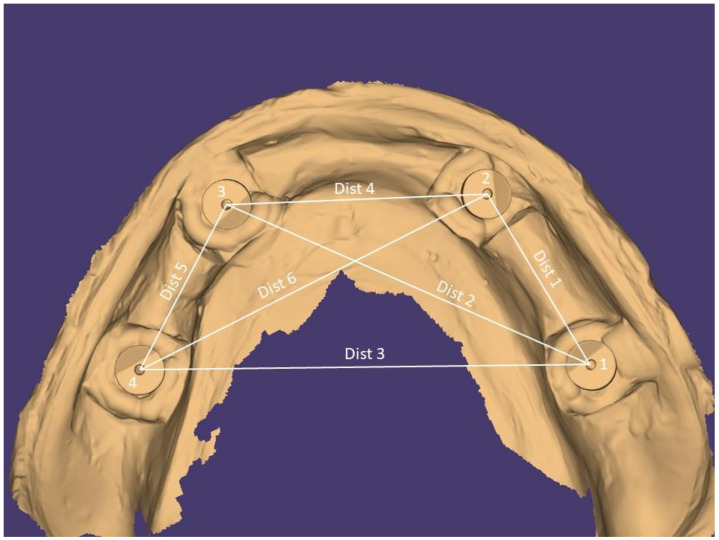
Digital impression with the scan posts and their centroids. Distances and number of each implant for the master model are shown.

The distances were assigned to two groups: the short-distance group (distances of adjacent scan posts during scan progression) D1–2, D2–3, D3–4, and the long-distance group (distances of the scan posts with non-adjacent sites in the arch) D1–3, D1–4, and D2–4 (Figure 2). The same operator (GK) conducted all measurements on the STL files of the 7 intraoral and 2 laboratory scanners. The centroid of each scan body was numerically defined and their respective distances were calculated by the same process that was described for the master model, again by use of PC-DMIS dedicated measurement software in offline mode.

Therefore, 10 scans from each of the tested IOSs were obtained by one operator (DS) with the same digital scanning protocol. Each scanner was calibrated according to manufacturer’s instructions. Starting from the distal area of the most distal implant on the right occlusal surface capturing images occlusally until the left distal implant turning to the buccal of the left implant and capturing until the buccal area of the right distal implant and finishing by capturing the lingual side of the model and the scan bodies. All scans were obtained in a 3-month period (October–December 2019) with the latest acquisition software available at that time for each digital scanner. The scans were obtained at room temperature (20 + 1 °C) with the same conditions regarding light and humidity (50%). Each scan was saved as a Standard Tessellation Language (STL) file, having 90 STL files from the 9 digital scanners tested. Laboratory scanners were used according to manufacturer instructions.

Trueness (comparisons with the master model measured with CMM; *n* = 10) and precision (intragroup comparisons; *n* = 45) of the measurements, using the absolute values of these differences, were examined. Separate general linear models (LMs) were constructed, with the scanner and implant distances as fixed factors, as well as their interaction. Box-cox-transformed trueness and precision values were used to fulfil normality assumptions.

### 2.2. Statistical Analyses

All statistical analyses for trueness and precision for short and long distances were performed in R v.3.6.3 (R Development Core Team, Vienna, Austria, 2020). Statistical models were fitted with the lme4 package (v. 1.1-23) [26] and appropriate fit was ensued by evaluating the distribution of model residuals. To test the significance of model terms, analysis of variance (ANOVA) was used (car package v. 3.0-8) [26], with Type III Wald chi-square tests and *p*-values (significant level α = 0.05) obtained from the ANOVA summaries. For post hoc comparisons the emmeans function was used with the Tukey method of the emmeans package (v. 1. 5. 0) [27]. Power analysis (GPower v3.1; Franz Faul Universität, Düsseldorf, Germany) verified at least 0.8 power, confirming that the sample size of 10 impressions made per group was adequate.

## 3. Results

**Figure 3:**
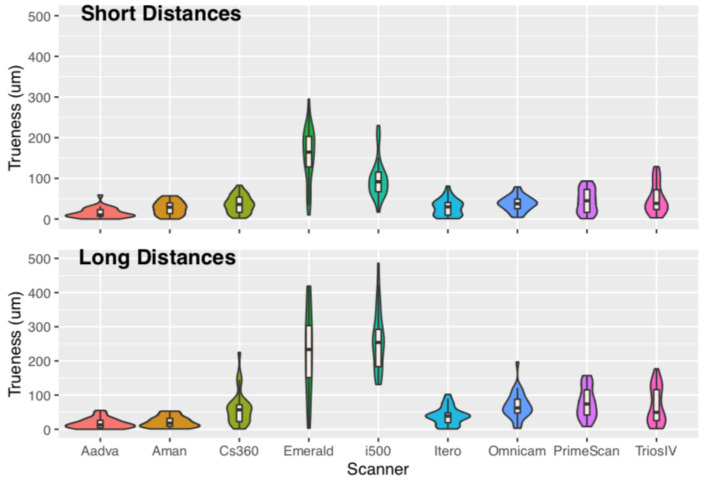
Linear distance mean error and comparison between different digital scanners for trueness.

**Figure 4:**
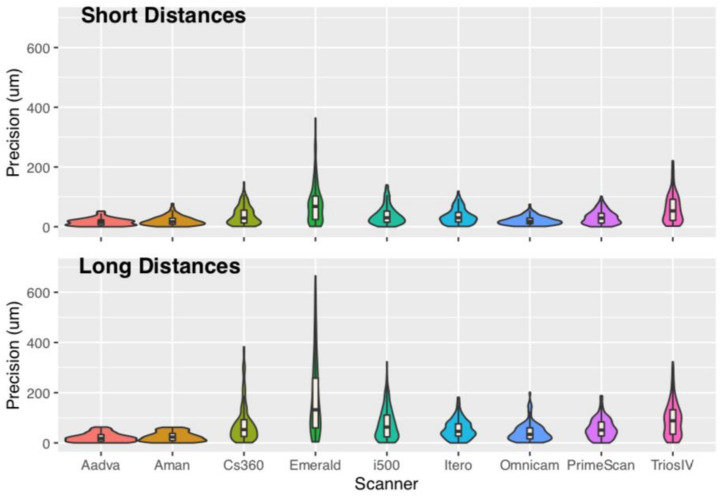
Linear distance mean error and comparison between different digital scanners for precision.

**Figure 5:**
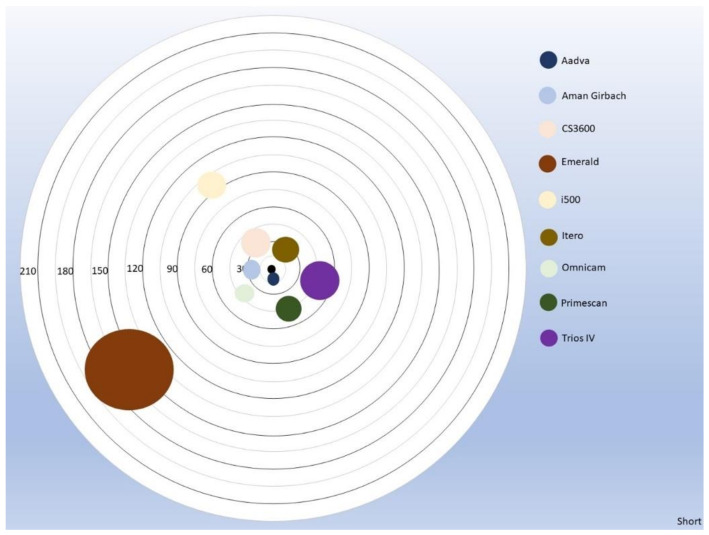
The distance of the circles to the center represents the “trueness” whereas the diameter of the circles represents “precision” for the short distances.

**Figure 6:**
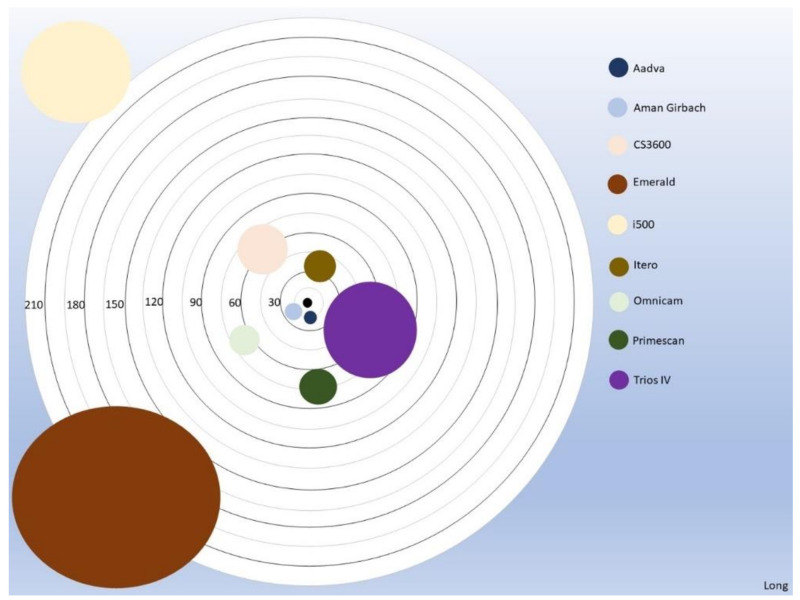
The distance of the circles to the center represents the “trueness” whereas the diameter of the circles represents “precision” for the long distances.

Analysis of variance showed that the factor scanner was significant for both trueness and precision. The factor distance was not significant; however, the factor scanner had an interaction with distance (Table 2 and Table 3). Descriptive statistic absolute values of trueness and precision for each distance were given as median with respective interquartile range (IQR) and mean with standard deviation (SD), with all values in μm (Table 4 and Table 5, Figure 3, Figure 4, Figure 5 and Figure 6). Mean values for trueness ranged from 15 μm (Aadva) to 157 μm (i500) regarding the short-distance group and −17 μm (Aadva) to 259 μm (i500) regarding the long-distance group. Precision values ranged from 16 μm (Aadva) to 78 μm (Emerald) regarding the short-distance group and 22 μm (Aadva) to 185 μm (Emerald) regarding the long-distance group (Table 4).

Statistical analysis showed that trueness and precision measurements of laboratory scanners and IOSs were significantly different from the master model measured with the CMM. The laboratory scanners showed significantly higher trueness and precision than all IOSs tested for the long-distance group. i500 (259 μm) and Emerald (236 μm) had the worst trueness results compared to all the other IOSs. Only IOS iTero had no different trueness than one of the laboratory scanners (Aman). For the long-distance group, precision differed among the IOSs and the least-precise seemed to be Emerald (185 μm) and statistically significant to all the other scanners (Table 5).

For the short distance, some IOSs were not different in trueness and precision compared to the laboratory scanners. Emerald had the worst trueness 158 μm, followed by i500 99 μm. All the other scanners showed variation with no statistically significant differences in terms of trueness (Table 4). In terms of precision, Emerald had the worst results, 185 μm, followed by TRIOS, 4 93 μm (Table 5).

## 4. Discussion

The null hypothesis was rejected since significant differences in the trueness and precision between the different intraoral and laboratory scanners exist. Since intraoral scanners use optical systems for recording geometry, it is expected that their accuracy and precision may be affected by the optical properties of the object being scanned [28,29]. Most of the studies evaluating IOS accuracy used dental stone or plaster models, metal scan bodies, or resin 3D-printed models. These materials have different optical properties than enamel, dentin, and restorative materials that may be found intraorally. Enamel seems to have higher refraction index than dentin [30]; also, enamel together with dentin, amalgam, and composite influence the overall accuracy of intraoral scanners. An in vitro study showed that dentin seems to be the most accurate substrate when scanned and enamel is the least accurate [31].

In this in vitro study, a type IV stone model was chosen to simulate a clinical case of an edentulous patient with four implants. A dentate model was not selected since we opted for a reference model simulating an edentulous patient with scan bodies of simple geometry, that allow for measurements across well-defined points, avoiding errors due to landmark identification. Many in vitro studies conduct a superimposition of the reference STL model with the STL models produced from the scanners [32]. This alignment is guided by the best-fit algorithm of a reverse engineering software that allows surfaces to fully overlap. Every overlapping image aligned with the best-fit algorithm could lead to uncertainty. Scanning longer spans would require more stitching of images and possibly lead to more uncertainties [16,33,34]. Additionally, the superimposition technique shows the deflection of each scan post or implant in comparison to the reference model, and the resulting number shows indirectly how much the distance of two adjacent or cross-arch implants has changed. In order to avoid errors from the algorithm during the superimposition in this study, the linear evaluation approach was selected. It is a proven method for specific geometries as the scan bodies and their centroids [20,35,36]. Future metrological studies should compare the results of direct measurements vs. the superimposition technique.

The basic requirement for an impression of the complete arch with implants is the choice of scan post. In this case, the Elos Medtech scan posts were adopted. PEEK scan posts with a titanium base seem to have good results in terms of accuracy in digital implant impression [37] and seem to show a potential clinical advantage in comparison with other scan posts as the digital impression of relatively flat and uncomplicated structure scan post seem to have lower deviations compared to other. [38]. During the period between the scans, the scan posts were never removed from the cast, as this could potentially result in a discrepancy and influence the results, as shown by Pan et al. [39].

An important issue is the use of the absolute distance differences for statistical analyses. By not using absolute values, negative and positive values in one scanner would neutralize each other and provide a lower mean. Additionally, scanners with the same absolute value but opposite signs would present as significantly different, even if the clinical effect would be exactly the same.

In the statistical results, we can see in the ANOVA (Table 2 and Table 3) that distance is not a significant factor for trueness and precision. However, there is an interaction between distance and scanner. Analyzing the results, we could summarize that there is a larger decrease in trueness and precision, as the distance between the scan bodies is increasing for IOS in comparison to the laboratory scanners. This is supported from other studies too [15,16].

In our study, both laboratory scanners were statistically different from the master model in trueness and precision. Since we know from the literature [15,40,41] that we can rely on them in order to scan the analog models and fabricate a fixed prosthesis, we should assume that their measured trueness and precision are within acceptable clinical levels, even though there are differences between them [42], especially when the inaccuracy of a conventional impression and model making as a prerequisite for laboratory scanners is considered.

From the group of intraoral scanners, Emerald showed the highest deviation in trueness and precision from all the other IOSs; other studies agree with our findings [4,43]. Sami et al. did not find clinical differences among the scanners when they scanned a polymer edentulous model with six hexagonal scan bodies, even if they used similar IOS [True Definition (3M Company, St. Paul, MN, USA), TRIOS (3Shape A/S, Copenhagen, Denmark), CEREC Omnicam (Sirona Dental Systems GmbH, Bensheim, Germany), Emerald Scanner (Planmeca Oy, Vantaa, Finland)], but the method chosen with superimposition of the digital casts [19] was different than the method used in the present study. Additionally, it is difficult to make direct comparisons between different studies as the date of release of each scanner is different and the software that the IOS used has been upgraded over the years.

All IOSs except Emerald in short distances and Emerald and i500 in long distances had trueness values lower than 150 μm, which is a proposed threshold for one historic implant system to achieve a clinical acceptable outcome [7,44]. These findings were in accordance with an in vivo study that showed IOSs being less accurate when the distance of the implants was increasing [45,46]. We do not know if this threshold is applicable to current internal-connection low-tolerance implant systems. The clinical approach to addressing misfit in metal superstructures involves using techniques, such as sectioning and soldering. However, for zirconia frameworks or monolithic zirconia superstructures, there is currently no solution for misfit, other than creating a new superstructure from a more accurate impression. One proposed alternative approach is to create a metal-bar substructure and then cement a zirconia superstructure onto it, which would help to resolve any misfit issues in the framework.

In our in vitro study, a mandibular model was used and this could be a limitation for our results [45]. In a clinical situation, where tissue mobility, humidity, temperature, and saliva exist, the outcome of the study could differ [47]. There are clinical reports presenting a successful completion of complete-arch cases treated with a fully digital protocol [48]. Further in vivo clinical studies should be carried out in the field. Comparison with stereophotogrammetry has favored the latter [49]. Continued evolvement of IOSs calls for new investigations of trueness and precision for newer devices in the future.

## 5. Conclusions

Within the limitations of the present in vitro study, we could conclude that:

1. Laboratory scanners have the best trueness and precision compared to all IOSs for long distances. Only iTero had comparable trueness with one laboratory scanner in short and long distances.
2. For short distances, CS360, Omnicam, Primescan, and TRIOS 4 had similar trueness to one laboratory scanner. From those, only Omnicam and Primescan had similar precision as the same laboratory scanner.
3. Most IOSs seem to work better in smaller distances and are less reliable in cross-arch distances.

## Figures and Tables

**Table 1:** List of intraoral and laboratory scanners.

Name	Manufacturer	Acquisition Technology	Output Files
Aadva	GC Corporation, Tokyo, Japan	High end dual camera system with structured blue led light	open STL or PLY data.
ceramill map 600	Amann Girrbach AG, Koblach, Austria	HD scan via 3D sensor with blue light technology	open STL or PLY data.
Cs3600	Carestream Health, Inc., Rochester, NY, USA	LED light scanner -Active Speed 3D Video	csz (proprietary format), ply and stl (open formats)
Emerald	Planmeca Oy, Vantaa, Finland	Red, green and blue lasers- Projected Pattern Triangulation	3oxz (proprietary format), ply and stl (open formats)
i500	Medit, Seoul, South Korea	3D in Motion Video Technology	obj, ply and stl (open formats)
iTero	Align Technologies Inc., Milpitas, CA, USA	Parallel Confocal Microscopy	3ds (proprietary format); ply and stl (open formats)
Omnicam	Sirona Dental Systems GmbH, Bensheim, Germany	Optical Triangulation and Confocal Microscopy	cs3, sdt, cdt, idt (proprietary format) with possibility to export .stl files (open format) with Cerec Connect^®^
PrimeScan	Sirona Dental Systems GmbH, Bensheim, Germany	High-resolution Sensors and Shortwave Light with Optical High Frequency Contrast Analysis for Dynamic Deep Scan (20 mm)	dxd (proprietary format) with possibility to export .stl files (open format) with Cerec Connect^®^
Trios 4	3-Shape A/S, Copenhagen, Denmark	Confocal Microscopy and Ultrafast Optical Scanning	dcm (proprietary format), with possibility to export stl files (open formats) with Trios on Dental Desktop^®^

**Table 2:** ANOVA results for trueness.

Response: Abs Difference Trueness				
	Sum of Squares	df	F Value	*p*-Value
(Intercept)	1.670	1	1414.940	<0.001
Scanner	43.686	8	662.080	<0.001
Distance Group	0.0007	1	0.0804	0.777
Scanner: Distance Group	0.4028	8	61.041	<0.001
Residuals	43.054	522		

**Table 3:** ANOVA results for precision.

Response: Abs Difference Precision				
	Sum of Squares	df	F Value	*p*-Value
(Intercept)	9.0063	1	775.760	<0.001
Scanner	6.9022	8	74.315	<0.001
Distance Group	0.0349	1	3.010	0.083
Scanner: Distance Group	0.6018	8	6.479	<0.001
Residuals	28.0026	2412		

**Table 4:** Descriptive statistic values of absolute median, interquartile range (IQR), mean, and standard deviation (SD) for trueness (μm). Groups with the same letter in the same distance are not statistically different.

Scanner	Distance	AbsT Median	AbsT IQR	AbsT Mean (SD)	Statistical Grouping
Aadva	short	10.0	16.0	15.0 (13)	A
Aman	short	29.0	26.3	27.1 (17)	AB
Cs3600	short	36.0	37.8	36.5 (21)	B
Emerald	short	164.5	75.3	157.7 (67)	D
i500	short	91.5	48.5	98.5 (48)	C
iTero	short	30.0	31.3	28.9 (20)	AB
Omnicam	short	37.5	23.5	38.0 (18)	B
Primescan	short	45.0	56.5	45.2 (29)	B
Trios 4	short	38.5	48.8	49.6 (36)	B
Aadva	long	13.0	21.8	17.2 (15)	A
Aman	long	18.0	22.8	21.6 (16)	AB
Cs3600	long	57.0	49.3	61.4 (49)	CD
Emerald	long	233.5	152.5	226.3 (120)	E
i500	long	254.0	109.3	259.7 (87)	E
iTero	long	38.5	28.3	39.0 (26)	BC
Omnicam	long	62.5	40.5	68.7 (39)	D
Primescan	long	74.0	73.8	77.4 (45)	D
Trios 4	long	49.5	90.8	69.3 (53)	CD

**Table 5:** Descriptive statistic values of median, interquartile range (IQR), mean, and standard deviation (SD) for precision (μm). Groups with the same letter in the same distance are not statistically different.

Scanner	Distance	AbsT Median	AbsT IQR	AbsT Mean (SD)	Statistical Grouping
Aadva	short	15.0	17.0	16.2 (12)	A
Aman	short	17.0	20.0	20.0 (15)	AB
Cs3600	short	29.0	43.0	37.9 (31)	C
Emerald	short	68.0	79.0	78.2 (67)	D
i500	short	30.0	37.5	38.5 (21)	C
iTero	short	30.0	32.0	35.1 (36)	C
Omnicam	short	18.0	19.0	20.6 (14)	AB
PrimeScan	short	29.0	33.0	31.3 (23)	BC
Trios 4	short	53.0	71.0	61.5 (48)	D
Aadva	long	18.0	26.0	22.1 (17)	A
Aman	long	23.0	28.5	25.4 (18)	A
Cs3600	long	53.0	67.00	78.3 (79)	CD
Emerald	long	132.0	198.5	185.2 (154)	E
i500	long	63.0	87.0	75.7 (63)	C
iTero	long	46.0	49.0	54.8 (38)	BC
Omnicam	long	34.0	44.0	42.6 (36)	B
PrimeScan	long	51.0	56.0	57. 4 (40)	BC
Trios 4	long	88.0	97.5	93.1 (68)	D

## Data Availability

The data presented in this study are available on request from the corresponding author.

## References

[1] Joda T., Brägger U. (2016). Patient-centered outcomes comparing digital and conventional implant impression procedures: A randomized crossover trial. Clin. Oral Implants Res..

[2] Lee S.J., Macarthur R.X., Gallucci G.O. (2013). An evaluation of student and clinician perception of digital and conventional implant impressions. J. Prosthet. Dent..

[3] Gjelvold B., Chrcanovic B.R., Korduner E.K., Collin-Bagewitz I., Kisch J. (2016). Intraoral digital impression technique compared to conventional impression technique. A randomized clinical trial. J. Prosthodont..

[4] Mangano F.G., Admakin O., Bonacina M., Lerner H., Rutkunas V., Mangano C. (2020). Trueness of 12 intraoral scanners in the full-arch implant impression: A comparative in vitro study. BMC Oral Health.

[5] Kim R.J.-Y., Benic G.I., Park J.-M. (2019). Trueness of digital intraoral impression in reproducing multiple implant position. PLoS ONE.

[6] Rutkūnas V., Gečiauskaitė A., Jegelevičius D., Vaitiekūnas M. (2017). Accuracy of digital implant impressions with intraoral scanners. A systematic review. Eur. J. Oral Implantol..

[7] (1994). Accuracy (Trueness and Precision) of Measurement Methods and Results—Part 1: General Principles and Definitions.

[8] (1994). Accuracy (Trueness and Precision) of Measurement Methods and Results—Part 2: Basic Method for the Determination of Repeatability and Reproducibility of a Standard Measurement Method.

[9] Joda T., Bragger U., Zitzmann N.U. (2019). CAD/CAM implant crowns in a digital workflow: Five-year follow-up of a prospective clinical trial. Clin. Implant Dent. Relat. Res..

[10] Papaspyridakos P., Gallucci G.O., Chen C.J., Hanssen S., Naert I., Vandenberghe B. (2016). Digital versus conventional implant impressions for edentulous patients: Accuracy outcomes. Clin. Oral Implants Res..

[11] Amin S., Weber H.P., Finkelman M., El Rafie K., Kudara Y., Papaspyridakos P. (2017). Digital vs. conventional full-arch implant impressions: A comparative study. Clin. Oral Implants Res..

[12] Alikhasi M., Siadat H., Nasirpour A., Hasanzade M. (2018). Three-Dimensional Accuracy of Digital Impression versus Conventional Method: Effect of Implant Angulation and Connection Type. Int. J. Dent..

[13] Ribeiro P., Herrero-Climent M., Díaz-Castro C., Ríos-Santos J.V., Padrós R., Mur J.G., Falcão C. (2018). Accuracy of Implant Casts Generated with Conventional and Digital Impressions-An In Vitro Study. Int. J. Environ. Res. Public Health.

[14] Tan M.Y., Yee S.H.X., Wong K.M., Tan Y.H., Tan K.B.C. (2019). Comparison of Three-Dimensional Accuracy of Digital and Conventional Implant Impressions: Effect of Interimplant Distance in an Edentulous Arch. Int. J. Oral Maxillofac. Implants.

[15] Flügge T.V., Att W., Metzger M.C., Nelson K. (2016). Precision of Dental Implant Digitization Using Intraoral Scanners. Int. J. Prosthodont..

[16] Giménez B., Özcan M., Martínez-Rus F., Pradíes G. (2014). Accuracy of a digital impression system based on parallel confocal laser technology for implants with consideration of operator experience and implant angulation and depth. Int. J. Oral Maxillofac. Implants.

[17] Mangano F.G., Veronesi G., Hauschild U., Mijiritsky E., Mangano C. (2016). Trueness and Precision of Four Intraoral Scanners in Oral Implantology: A Comparative in Vitro Study. PLoS ONE.

[18] Imburgia M., Logozzo S., Hauschild U., Veronesi G., Mangano C., Mangano F.G. (2017). Accuracy of four intraoral scanners in oral implantology: A comparative in vitro study. BMC Oral Health.

[19] Sami T., Goldstein G., Vafiadis D., Absher T. (2020). An in vitro 3D evaluation of the accuracy of 4 intraoral optical scanners on a 6-implant model. J. Prosthet. Dent..

[20] Papazoglou E., Wee A.G., Carr A.B., Urban I., Margaritis V. (2020). Accuracy of complete-arch implant impression made with occlusal registration material. J. Prosthet. Dent..

[21] Michalakis K.X., Asar N.V., Kapsampeli V., Magkavali-Trikka P., Pissiotis A.L., Hirayama H. (2012). Delayed linear dimensional changes of five high strength gypsum products used for the fabrication of definitive casts. J. Prosthet. Dent..

[22] (2009). Geometrical Product Specifications (GPS)—Acceptance and Reverification Tests for Coordinate Measuring Machines (CMM)—Part 2: CMMs Used for Measuring Linear Dimensions.

[23] (2010). Geometrical Product Specifications (GPS)—Acceptance and Reverification Tests for Coordinate Measuring Machines (CMM)—Part 5: CMMs Using Single and Multiple Stylus Contacting Probing Systems.

[24] (2001). Geometrical Product Specifications (GPS)—Acceptance and Reverification Tests for Coordinate Measuring Machines (CMM)—Part 6: Estimation of Errors in Computing Gaussian Associated Features.

[25] (1989). Guide to Assessment of Position, Size and Departure from Nominal Form of Geometric Features.

[26] Bates D., Mächler M., Bolker B., Walker S. (2015). Fitting Linear Mixed-Effects Models Using lme4. J. Stat. Softw..

[27] Lenth R. (2019). Emmeans: Estimated Marginal Means, aka Least-Squares Means. https://CRAN.Rproject.org/package=emmean.

[28] Lim J.H., Mangal U., Nam N.E., Choi S.H., Shim J.S., Kim J.E. (2021). A Comparison of Accuracy of Different Dental Restorative Materials between Intraoral Scanning and Conventional Impression-Taking: An In Vitro Study. Materials.

[29] Dutton E., Ludlow M., Mennito A., Kelly A., Evans Z., Culp A., Kessler R., Renne W. (2020). The effect different substrates have on the trueness and precision of eight different intraoral scanners. J. Esthet. Restor. Dent..

[30] Meng Z., Yao X.S., Yao H., Liang Y., Liu T., Li Y., Wang G., Lan S. (2009). Measurement of the refractive index of human teeth by optical coherence tomography. J. Biomed. Opt..

[31] Bocklet C., Renne W., Mennito A., Bacro T., Latham J., Evans Z., Ludlow M., Kelly A., Nash J. (2019). Effect of scan substrates on accuracy of 7 intraoral digital impression systems using human maxilla model. Orthod. Craniofac. Res..

[32] Wulfman C., Naveau A., Rignon-Bret C. (2020). Digital scanning for complete-arch implant-supported restorations: A systematic review. J. Prosthet. Dent..

[33] Güth J.F., Edelhoff D., Schweiger J., Keul C. (2016). A new method for the evaluation of the accuracy of full-arch digital impressions in vitro. Clin. Oral Investig..

[34] Braian M., Wennerberg A. (2019). Trueness and precision of 5 intraoral scanners for scanning edentulous and dentate complete-arch mandibular casts: A comparative in vitro study. J. Prosthet. Dent..

[35] Ender A., Attin T., Mehl A. (2016). In vivo precision of conventional and digital methods of obtaining complete-arch dental impressions. J. Prosthet. Dent..

[36] Mehl A., Reich S., Beuer F., Güth J.F. (2021). Accuracy, trueness, and precision—A guideline for the evaluation of these basic values in digital dentistry. Int. J. Comput. Dent..

[37] Revilla-León M., Fogarty R., Barrington J.J., Zandinejad A., Özcan M. (2020). Influence of scan body design and digital implant analogs on implant replica position in additively manufactured casts. J. Prosthet. Dent..

[38] Motel C., Kirchner E., Adler W., Wichmann M., Matta R.E. (2020). Impact of Different Scan Bodies and Scan Strategies on the Accuracy of Digital Implant Impressions Assessed with an Intraoral Scanner: An In Vitro Study. J. Prosthodont..

[39] Pan Y., Tam J.M.Y., Tsoi J.K.H., Lam W.Y.H., Pow E.H.N. (2020). Reproducibility of laboratory scanning of multiple implants in complete edentulous arch: Effect of scan bodies. J. Dent..

[40] Nulty A.B. (2021). A Comparison of Full Arch Trueness and Precision of Nine Intra-Oral Digital Scanners and Four Lab Digital Scanners. Dent. J..

[41] Kang B.-H., Son K., Lee K.-B. (2020). Accuracy of Five Intraoral Scanners and Two Laboratory Scanners for a Complete Arch: A Comparative In Vitro Study. Appl. Sci..

[42] Nowak R., Wesemann C., Robben J., Muallah J., Bumann A. (2017). An in-vitro study comparing the accuracy of? full-arch casts digitized with desktop scanners. Quintessence Int..

[43] Diker B., Tak Ö. (2020). Comparing the accuracy of six intraoral scanners on prepared teeth and effect of scanning sequence. J. Adv. Prosthodont..

[44] Jemt T. (1991). Failures and complications in 391 consecutively inserted fixed prostheses supported by Brånemark implants in edentulous jaws: A study of treatment from the time of prosthesis placement to the first annual checkup. Int. J. Oral Maxillofac. Implants.

[45] Orejas-Perez J., Gimenez-Gonzalez B., Ortiz-Collado I., Thuissard I.J., Santamaria-Laorden A. (2022). In Vivo Complete-Arch Implant Digital Impressions: Comparison of the Precision of Three Optical Impression Systems. Int. J. Environ. Res. Public Health.

[46] Alpkılıç D.Ş., Değer S.İ. (2022). In Vitro Comparison of the Accuracy of Conventional Impression and Four Intraoral Scanners in Four Different Implant Impression Scenarios. Int. J. Oral Maxillofac. Implants.

[47] Knechtle N., Wiedemeier D., Mehl A., Ender A. (2022). Accuracy of digital complete-arch, multi-implant scans made in the edentulous jaw with gingival movement simulation: An in vitro study. J. Prosthet. Dent..

[48] Manazza F., La Rocca S., Nagni M., Chirico L., Cattoni F. (2021). A simplified digital workflow for the prosthetic finishing of implant rehabilitations: A case report. J. Biol. Regul. Homeost. Agents.

[49] Kosago P., Ungurawasaporn C., Kukiattrakoon B. (2022). Comparison of the accuracy between conventional and various digital implant impressions for an implant-supported mandibular complete arch-fixed prosthesis: An in vitro study. J. Prosthodont..

